# Effect of Ambient Environment on Laser Reduction of Graphene Oxide for Applications in Electrochemical Sensing

**DOI:** 10.3390/s23188002

**Published:** 2023-09-21

**Authors:** Abdullah A. Faqihi, Neil Keegan, Lidija Šiller, John Hedley

**Affiliations:** 1 School of Engineering, Newcastle University, Newcastle upon Tyne NE1 7RU, UK; eng.fakihi@gmail.com (A.A.F.); lidija.siller@newcastle.ac.uk (L.Š.); 2 Department of Industrial Engineering, College of Engineering, Jazan University, Jazan 45142, Saudi Arabia; 3 Translational and Clinical Research Institute, Newcastle upon Tyne NE1 7RU, UK; neil.keegan@newcastle.ac.uk

**Keywords:** reduced graphene oxide, electrochemical sensors, sensor fabrication, laser reduction, cyclic voltammetry

## Abstract

Electrochemical sensors play an important role in a variety of applications. With the potential for enhanced performance, much of the focus has been on developing nanomaterials, in particular graphene, for such sensors. Recent work has looked towards laser scribing technology for the reduction of graphene oxide as an easy and cost-effective option for sensor fabrication. This work looks to develop this approach by assessing the quality of sensors produced with the effect of different ambient atmospheres during the laser scribing process. The graphene oxide was reduced using a laser writing system in a range of atmospheres and sensors characterised with Raman spectroscopy, XPS and cyclic voltammetry. Although providing a slightly higher defect density, sensors fabricated under argon and nitrogen atmospheres exhibited the highest average electron transfer rates of approximately 2 × 10^−3^ cms^−1^. Issues of sensor reproducibility using this approach are discussed.

## 1. Introduction

Electrochemical sensors play an important role in our day-to-day lives. Their sensing schemes are based on several means of electrochemical recognition, such as coulometry, amperometry, potentiometry and voltammetry [1]. Through electrochemistry, researchers can examine diverse systems. In particular, electrochemistry can help provide a better understanding of the role of catalysts and catalytic efficiency, as well as chemical reduction and oxidation (redox) processes, assemble or synthesise materials, examine the efficiency and functions of catalysts and determine the reversibility of a chemical process [2]. They are used in many areas and have found applications in environmental monitoring, food monitoring and medical diagnostics [3,4,5,6]. A range of materials may be used for the electrodes [7]; in particular, carbon is a popular choice because of the ability to easily functionalise this material [8,9]. The techniques of screen printing and inkjet printing [10] are widely used in the production of carbon-based electrodes.

There have been significant developments in the synthesis, characterisation and processing, as well as application, of nanomaterials utilised in the design of electrochemical sensors [11]. In the development of electrochemical biosensors, there has been considerable focus on nanostructured carbon-based materials [12], including carbon nanotubes [13,14], carbon quantum dots [15,16] and graphene [17,18]. Graphene materials have enormous potential because of their exceptional electrochemical and mechanical strength, good adsorption performance and flexibility [19,20]. Graphene-based materials have been reported to possess a surface area of 2630 m^2^g^−1^, and this property is useful for sensing applications where the surface area is of importance for the efficiency of the device [21,22]. Moreover, with a framework that can potentially be rich in oxygen and other defects, graphene can be developed as nanocomposites for a range of applications [23].

Methods for the production of graphene can be divided into bottom-up approaches such as chemical vapour deposition [24,25,26] and epitaxial growth on silicon carbide [27,28] or top-down approaches, which include the liquid phase and the chemical exfoliation of graphite [29,30,31]. Although top-down approaches only produce graphene flakes of sizes ranging from 100s of nm to 10s of µm, these methods are easily scalable allowing for large production volumes. Chemical approaches produce graphene oxide flakes, which require reduction to transform back into graphene. This can be conducted either chemically, for example, with the use of hydrazine hydrate [23], or thermally at temperatures ranging from 500 °C to over 1100 °C to expel the oxygen atoms [32]. The use of a laser to locally heat graphene oxide has shown great promise because of its ability to both perform reduction and patterning of the material simultaneously. The final reduced product is termed reduced graphene oxide (rGO) with the flakes showing an increased number of defects to the carbon lattice compared to pristine graphene flakes. These defects, however, can be advantageous depending on the application of the material, for example, allowing for increased bonding sites for functionalisation [23]. Reduced graphene oxide material is becoming prominent as a useful and promising material for graphene-based applications, such as sensors [17,33], supercapacitors [34,35], and electrodes [36,37]. This turn towards rGO is mainly because of its lower cost, hydrophilic affinity, ease of handling, and the possibility of the mass production of devices [38].

A number of researchers have looked to correlate the laser reduction parameters with the properties of the resulting rGO film. Furio et al. [39] examined how the wavelength and intensity of the light used in the reduction process affected the wettability, electrical and transparency properties of the reduced graphene oxide films. Their work demonstrated higher power densities correlated to lower sheet resistance films, achieving values as low as 1300 Ωsq^−1^ using a CO_2_ laser. Komarov et al. [40] compared reduced graphene oxide films produced using low-cost lasers, concluding that shorter wavelengths produce better quality films. Mortazavi et al. [41] also demonstrated the advantages of a shorter wavelength using a 10 ns pulsed KrF excimer laser operating at a wavelength of 248 nm to thermally reduce graphene oxide, achieving the greatest reduction at laser fluences of 10 mJcm^−2^ to 30 mJcm^−2^ before the onset of ablation. Using a 780 nm femtosecond laser, Wan et al. [42] investigated the effect of laser power and scan speed on the reduction of GO. Their research showed that these two parameters can be utilised to separately control the level of conversion of sp^3^ into sp^2^ carbon and the amount of oxygen content, thus providing the ability to fine tune the material to specific applications. Naik et al. [43] utilised a 532 nm Millennia CW laser to conduct controlled reduction at power densities up to 17 Wcm^−2^ corresponding to a measured temperature of 200 °C. It was deduced that beyond this value, the heat generated from the reduction process also contributes to the heating of the surface resulting in a significant increase in the surface temperature. Using a 1064 nm picosecond laser and correlating this with a temperature dynamics simulation, Trusovas et al. [44] demonstrated that temperatures in excess of 1400 °C are achievable when energy densities of 0.32 Jcm^−2^ are used, whilst Orekhov et al. [45] showed, through a combination of experiment and simulation, that C-C bond formation and defect annealing are favourable at temperatures above 3000 K. A comprehensive review of the use of lasers for graphene oxide modification is given by Trusovas et al. [46].

This work aims to further develop the fabrication of electrochemical biosensors based on laser-scribed rGO electrodes, which have currently shown tremendous potential because of their large surface area and porous structure, with high charge transfer capabilities [47]. Originally developed for supercapacitor production [48], the technique involves the use of a simple LightScribe DVD drive with a laser using a wavelength of 788 nm and power of 5 mW to pattern and thermally reduce GO contained on the surface of a DVD disk [49]. The significant advantages of the LightScribe approach in GO reduction is that the process is simple to set up. The one-step patterning method can be performed in any laboratory that has basic computer facilities. However, the reduction process is performed under ambient atmospheric conditions and is, therefore, susceptible to the effects of additional oxygen being present during the process. Therefore, as highlighted by Pei et al. [32], reduction should take place in an inert or ideally reducing atmosphere [50]. This work aims to further develop this laser-scribing technique by examining the effects of conducting the laser reduction process under a range of controlled atmospheres with the aim of producing optimised electrodes for electrochemical sensing. The produced sensors are characterised via Raman, XPS and cyclic voltammetry measurements.

This paper is organized as follows. Leading on from this introduction, this paper describes the materials and hardware used to fabricate and, subsequently, characterise the sensors followed by a presentation and discussion of the results. Recommendations for future work are then suggested.

## 2. Materials and Methods

### 2.1. Hardware

**Figure 1:**
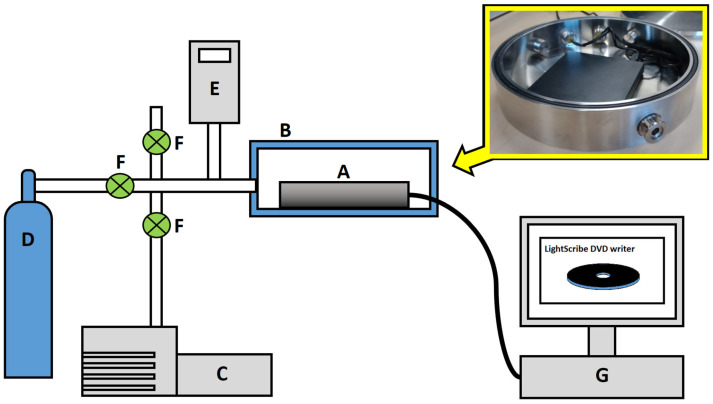
Schematic of the hardware setup used for sensor fabrication. (A) A LightScribe DVD writer is contained within (B) an enclosed chamber. The gas type and pressure are controlled via (C) a rotary vane vacuum pump, (D) gas cylinders, (E) pressure gauge and (F) stop values. (G) A PC is used to control the image writing process.

A schematic of the experimental setup used for the fabrication of the electrochemical sensors is shown in Figure 1. The main components are:

- A LightScribe DVD writer connected to a PC. The LightScribe is a direct disc-labelling system that burns a user-defined image on the nondata side of a LightScribe DVD [51];
- A custom-made, stainless-steel environmental chamber with electrical feedthroughs incorporated into the chamber wall allowing for the operation of the enclosed LightScribe system;
- A gas line consisting of a vacuum pump, a pressure sensor and any required gas cylinders.

### 2.2. Fabrication Process

The first step in the fabrication of the sensors involved cutting a DVD-sized annulus shape out of 115 µm thick polyethylene terephthalate (PET) film substrate (Viking Office Depot, Leicester, UK) with an area of 1 × 10^4^ mm^2^. The film was then attached to the top (label) side of a LightScribe DVD using 3M adhesive spray. Graphene oxide (GO) solution, obtained from the University of California at Los Angeles, was diluted in distilled water to a concentration of 75 mg/mL (as recommended in previous work [17]) and shaken for a 5 min period. Then, 15 mL of this GO solution was drop cast onto the PET substrate and, subsequently, left to dry for 48 h at room temperature in a dust-free environment.

The DVD containing the dried GO film was inserted into the LightScribe DVD writer. This writer was then placed into the vacuum chamber, and the corresponding cables were connected. If an atmosphere other than air was to be used, the chamber was evacuated down to a pressure of less than 1 mbar and refilled with the required atmospheric gas. The LightScribe Template Labeller software (Hewlett-Packard, Palo Alto, CA, USA, version 1.18.27.10) was then used to write a continuous track onto the GO film. In order to yield a well-designed sensing electrode, the pattern was scribed for 10 cycles onto the surface, with each cycle taking 15 min to complete.

For the atmospheres of air, pure nitrogen and pure argon, a pressure of 1 bar, 0.5 bar and 0.5 bar was used, respectively. The reduced pressures for nitrogen and argon ensured that the chamber lid was firmly held in place because of the atmospheric pressure. In the case of a vacuum environment, early attempts at laser scribing simply ablated the GO from the surface because of the lack of surface pressure. Therefore, for these conditions, laser heating was initially performed at atmospheric pressure to remove the majority of the oxygen atoms before finally being heated under vacuum conditions of less than 1 mbar.

**Figure 2:**
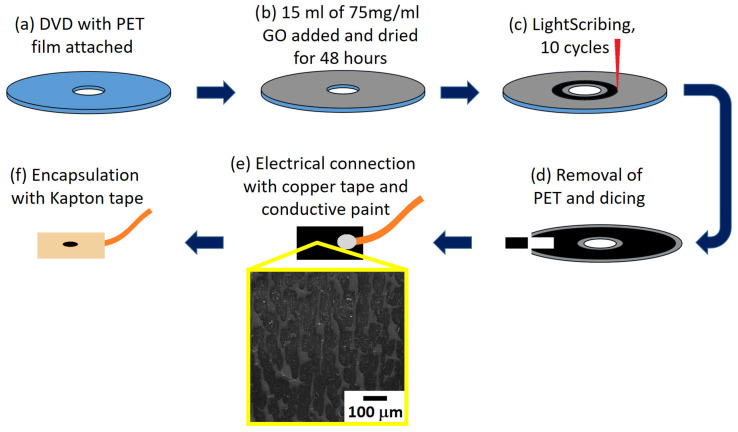
Fabrication of the rGO electrochemical sensors: (**a**) PET film is glued onto a LightScribe DVD; (**b**) 15 mL of GO solution is added and left to dry; (**c**) DVD is placed within a chamber-enclosed LightScribe DVD writer, the required atmosphere is set, and the electrode pattern is written onto the GO film reducing it to the darker coloured rGO; (**d**) upon completion, the PET film is removed and cut into corresponding sensors; (**e**) copper tape is attached to enable an electrical connection; (**f**) sensor is encapsulated in Kapton tape with a 3 mm diameter sensing window. The inset is an SEM image of the rGO surface.

It should be noted that there was some inconsistency in the LightScribe’s process, where on occasions it could be seen that the scribing process was ineffective. Upon completion of each scribing process, the disk was removed from the chamber, and the reduction process was visually inspected for consistency. A random set of well-scribed areas was cut from the PET film for testing. Individual sensors were attached to copper tape (RS Components Ltd., Corby, UK). Silver conductive paint (RS Components Ltd., Corby, UK) was used to ensure a good electrical contact between the sensor and tape. A 3 mm diameter hole was punched into a piece of Kapton tape (RS Components Ltd., Corby, UK), and then this piece of tape was used to enclose the sensor and copper tape. This ensured that only a well-defined conductive area was presented to the solution during the CV measurements. Figure 2 summarises the production process for the sensors.

### 2.3. Characterisation

#### 2.3.1. SEM

The fabricated samples were firstly assessed using a Jeol JSM 5610LV SEM machine (Joel (UK) Ltd., Welwyn Garden City, UK). The SEM images provide a visual verification of the arrangement of the graphene flakes and their topology after the reduction process.

#### 2.3.2. Raman Spectroscopy

Raman spectra of graphene and graphene oxide sheets may be used to determine its atomic structure, number of layers and any disorders. The Raman peak shape, position and intensity can all be used in the investigation of these features [52]. Two primary features appear in a graphene spectrum at wavenumbers of approximately 1580 cm^−1^ and 2690 cm^−1^ corresponding to the G and 2D bands, respectively. The position and broadness of the 2D band can be used in the calculation of the number of graphene layers present [53,54]. The faults and chemical composition of the graphene layer, particularly in the case of reduced graphene oxide, which still contains a relatively large number of oxygen bonds compared to pristine graphene, results in an additional peak at approximately 1340 cm^−1^, attributed to the D band, and a possible shift in the G band [55]. The intensity and area ratios of the D to G bands in a graphene sheet indicates the number of structural flaws present in the graphene material [56].

Raman spectra of the fabricated samples were obtained using a Horiba Jobin Yvon HR800 Raman spectrometer with a 30 mW, 512 nm argon ion excitation laser. Light was focused and collected through a ×100 objective and passed through a spectrometer consisting of 150 µm slit width and 1800 gr/mm grating. Each scan consisted of 2 accumulations at 5 s per accumulation in the range 100 cm^−1^ to 3000 cm^−1^. A 521 cm^−1^ peak of silicon was used to calibrate the wavenumber scale. The MATLAB (Mathworks, version R2021b) resampling function was then used to linearise the wavenumber scale of the Raman data to 0.5 cm^−1^ increments allowing for the input of the data into CasaXPS software (version 2.3.25) for analysis. Three regions were selected for analysis: 1200 cm^−1^ to 1465 cm^−1^, 1465 cm^−1^ to 1700 cm^−1^ and 2550 cm^−1^ to 2800 cm^−1^ corresponding to the D, G and 2D regions, respectively. A Shirley background was subtracted from each region and then a Voigt profile (lineshape parameter GL(70)) fit to each peak. The peak position, area and intensity, with the latter being calculated using the MATLAB interp1 and max functions on the CasaXPS-generated fitted profiles, were determined for each of the peaks.

#### 2.3.3. X-ray Photoelectron Spectroscopy

XPS is a surface-sensitive technique that provides information about the oxidative state and chemical composition of the elements present [57]. Typically a survey scan is performed to identify the elements present, with gold being introduced into the sample set as a calibration standard for the binding energy. Detailed scans are then performed focusing on specific regions in the spectrum. For rGO samples, an XPS analysis is considered to be a beneficial method to confirm the levels of reduction and the quality of the precursor material [58,59]. In this work, survey scans were used to identify the initial and final abundance of carbon, oxygen and nitrogen. This was followed by detailed scans of the carbon peak. For high-quality graphene, a peak corresponding to C-C binding is expected, whilst defects/functional groups present themselves at a range of higher binding energies. Oxygen-related functional groups occur in the range 285 eV–289 eV [58], whilst components due to C-N bonding would be present in the range 285 eV–286 eV [60]. A list of expected binding energy ranges is provided in Appendix A, Table A1. Recent work by Smith et al. [61] demonstrates the potential complexity of deconvoluting the carbon peak, with even the C-C bonding showing potentially multiple features due to the sp^2^ bonding, sp^3^ bonding and defective carbon structures.

The XPS spectra were collected from the samples using a Thermo Scientific K-alpha X-ray Photoelectron Spectrometer™ (Thermo Scientific, East Grinstead, UK). The spectra were acquired using a monochromatic Al Kα X-ray source with an output energy of 1486.6 eV and a maximum X-ray beam spot size of 400 μm. A survey scan was first taken using a dwell time of 100 ms, pass energy of 150 eV and step size of 0.2 eV, followed by a detailed scan of the carbon peak (scan range of 279 eV–290 eV, step size of 0.05 eV, dwell time of 300 ms and pass energy of 40 eV). The data were then input into the CasaXPS software (version 2.3.25PR1.0) for analysis, and a Shirley background subtracted from each region was analysed. The elemental abundances were determined from the 274 eV–296 eV, 393 eV–407 eV and 525 eV–540 eV regions of the survey scan. From the detailed scan of the carbon peak, four asymmetric Lorentzian lineshape components (lineshape parameter LA(50)) were found to be the minimum number of components needed to significantly reduce the residuals of the fit. The binding energy of these profiles was calibrated against the gold reference peaks, with the area and peak position then determined for each component.

#### 2.3.4. Cyclic Voltammetry

The basic theory of cyclic voltammetry is provided in Appendix B. The electrochemistry of the prepared electrodes used for this work involved the utilisation of a three-electrode system that consisted of the produced LightScribe graphene electrode, a platinum counter electrode and a standard aqueous Ag/AgCl reference electrode. Electrochemical measurements were obtained using a potentiostat (Autolab PGSTAT12, Metrohm UK Ltd., Runcorn, UK). Outer sphere potentials were obtained using 1,1’-ferrocene dimethanol at a 1 mM concentration in 3 M potassium chloride solution, whilst inner sphere potentials were obtained using potassium ferricyanide at a 1 mM concentration in 3 M potassium chloride solution. The measurements were performed at 20 °C. For the CV measurements with each redox species, 3 replicas of each electrode were tested at a scan rate of 10 mVs^−1^. When potassium ferricyanide was used, the potential was cycled between −0.15 V and 0.60 V, whilst in the case of the 1,1′-ferrocene dimethanol redox species, the potential was cycled between 0.00 V and 0.60 V. In a final experiment, CV was performed at five different scan rates on an electrode from each batch at 10 mVs^−1^, 25 mVs^−1^, 50 mVs^−1^, 75 mVs^−1^ and 100 mVs^−1^.

## 3. Results and Discussion

### 3.1. SEM

**Figure 3:**
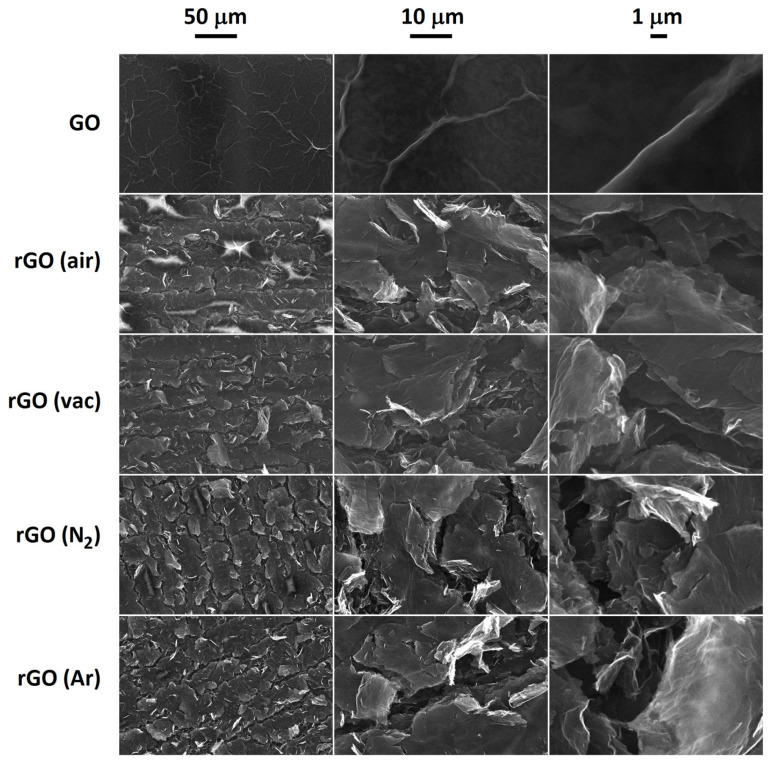
SEM images of the starting graphene oxide material (GO) and the reduced graphene oxide (rGO) produced via laser heating of the GO in air, under vacuum, nitrogen and argon atmospheres.

SEM images of the reduction process are shown in Figure 3. In the lower magnification rGO images, the tracking of the laser is clearly visible, whilst in the higher magnification images, the edges of the flakes are seen to expand out into single-layer graphene. The rGO material significantly expanded compared to the starting GO material; however, from a visual inspection, there was no obvious difference among the rGO produced in the four different atmospheres.

### 3.2. Raman

**Figure 4:**
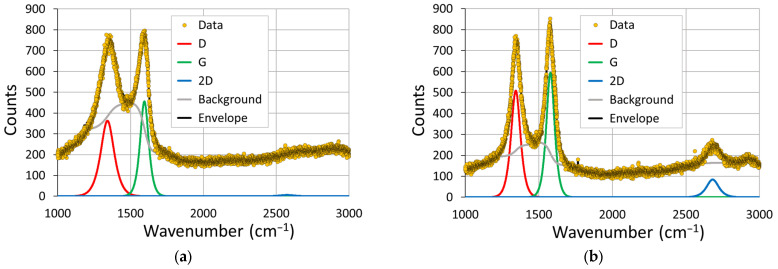
Examples of a (**a**) Raman scan of GO and a (**b**) Raman scan of rGO. Three components, corresponding to the D, G and 2D peaks of graphene were fit to each profile.

**Figure 5:**
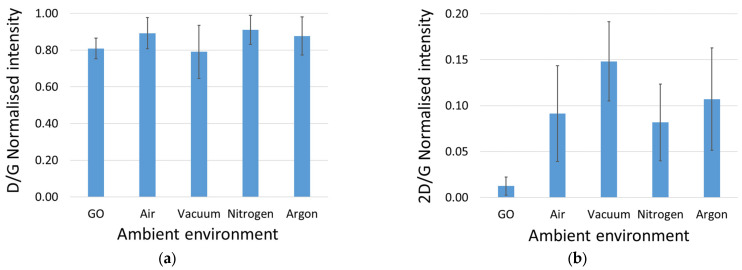
Relative intensities of the (**a**) D peak compared to the G peak and the (**b**) 2D peak compared to the G peak of 12 GO and 9 rGO (per environment) samples. The standard deviations of the measurements are indicated by the error bars.

Example Raman spectra from GO and rGO are shown in Figure 4 together with the fitted components, and the data analysis is summarised in Figure 5. A clear reduction of the graphene oxide was evident from the emergence of the 2D peak in the spectra, and the vacuum environment showed the highest ratio of 2D/G, with the three gaseous atmospheres performing similarly. The defect density appeared to effectively be unaltered by the reduction process, with the D/G ratio being maintained around the 0.8 to 0.9 value, with the vacuum environment demonstrating a slightly lower ratio. This appears to be consistent with the literature, where the reduction process of GO increases the defect density [62,63].

### 3.3. XPS

**Figure 6:**
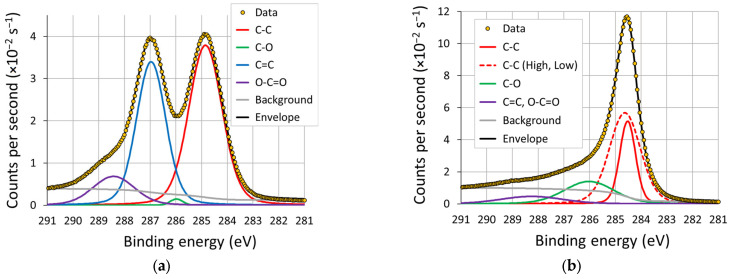
Examples of XPS spectra of the carbon peak from (**a**) GO and (**b**) rGO. Four components were fitted to each spectra and, subsequently, attributed to a specific bonding type.

Example XPS spectra from GO and rGO are shown in Figure 6. For rGO, the two dominant peaks were attributed to C-C bonds and C=C bonds with a shoulder at the high binding energy side, indicating a significant contribution for O-C=O bonds. The fourth component indicated some C-O bonding present, although the accuracy of the measurement of this component may be limited because of the dominance of the two adjacent peaks. The reduction showed a significant change in the profile of the spectra, with little evidence of the higher energy peaks. The C-C peak required two components to accurately fit the data; this is attributed to the potential of multiple C-C bonding scenarios, as described in Table A1. The C-O bond was now evident, and the remaining residuals at the higher frequency were attributed to any remaining C=O and O-C=O bonding.

**Figure 7:**
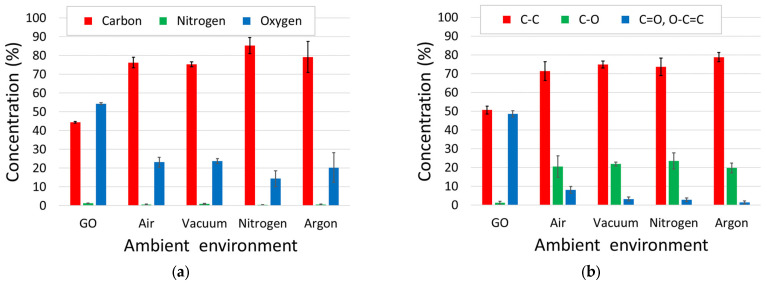
(**a**) Percentage abundance of carbon, nitrogen and oxidation obtained from XPS survey scans of the samples; (**b**) bond-type contribution to the C peak in each XPS spectra. The standard deviations of the measurements (n = 8 for GO, n = 6 for rGO (per environment)) are indicated by the error bars.

The results of the survey scans, shown in Figure 7a, indicated an increase in the relative carbon content of 30–40% and a corresponding decrease in the relative oxygen content. The nitrogen content was initially at 1.3%, which reduced to less than 1% in all cases. An analysis of the carbon peaks (the summarised results are provided in Figure 7b) highlight a shift in the remaining oxygen content from C=O bond formations to predominantly C-O bonding. This shift was more evident in the inert atmospheres (vacuum, nitrogen and argon) compared to laser reduction under normal atmospheric conditions. This loss of carboxylic groups whilst retaining hydroxyl groups suggests that temperatures of between 600 °C and 800 °C are achieved during the LightScribe process [64].

### 3.4. Cyclic Voltammetry

**Figure A1:**
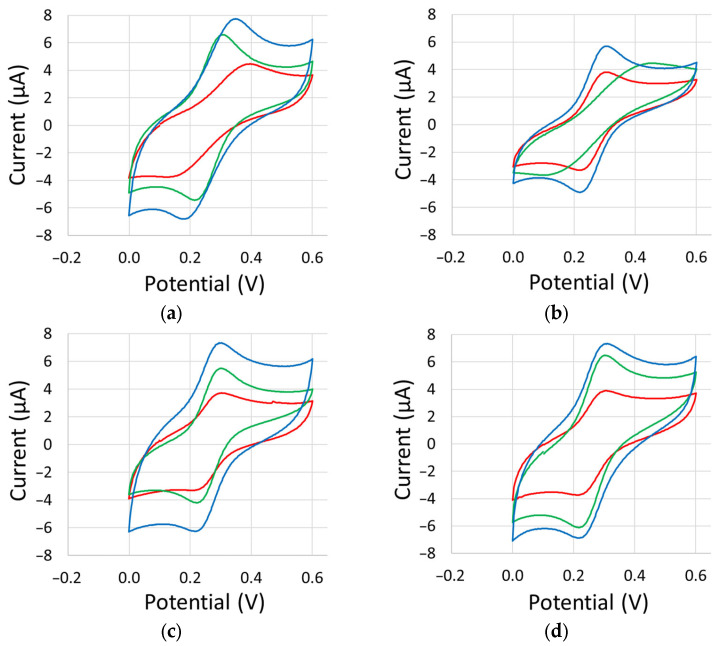
Outer sphere redox scans of 3 replicas of the sensors (plotted in red, green and blue respectively) fabricated in (**a**) air, (**b**) vacuum, (**c**) nitrogen, and (**d**) argon atmospheres.

**Figure A2:**
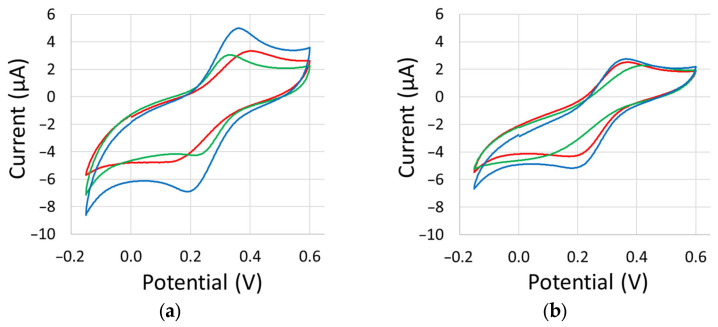

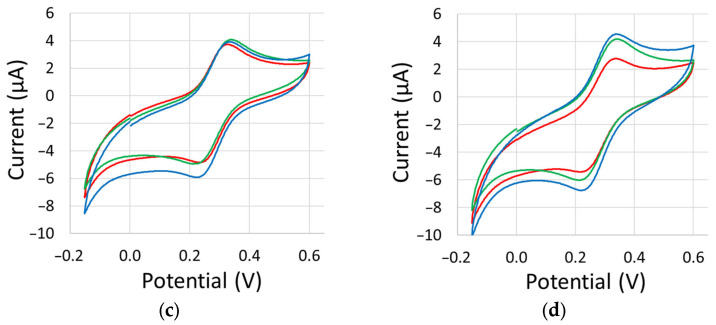
Inner sphere redox scans of 3 replicas of the sensors (plotted in red, green and blue respectively) fabricated in (**a**) air, (**b**) vacuum, (**c**) nitrogen, and (**d**) argon atmospheres.

Outer sphere redox scans for the 4 ambient atmospheres used are shown in Appendix B and Figure A1, whilst inner sphere redox scans are shown in Figure A2. Table 1 summarises the fitting of these curves. Under air or vacuum conditions, a larger variation was seen in the values. The minimum values achieved in each data set are provided to highlight the potential of a well-fabricated sensor.

**Figure 8:**
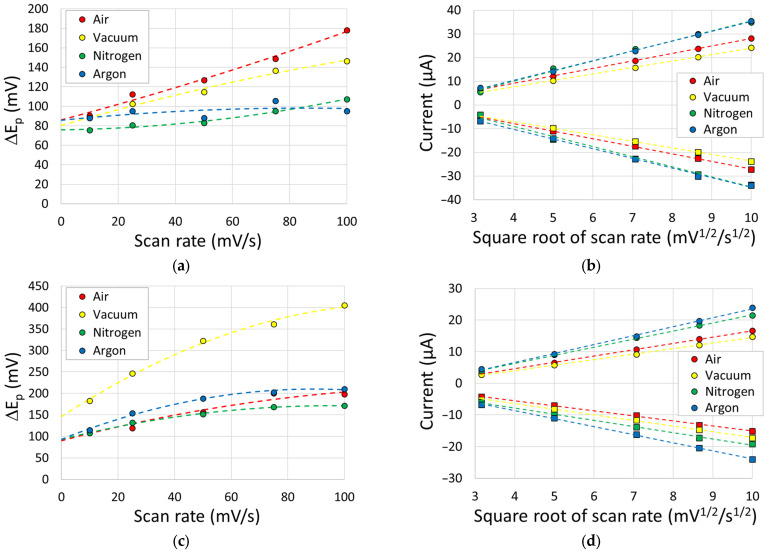
(**a**,**c**) Peak separation and (**b**,**d**) peak anodic (circle) and cathodic (square) currents for a range of cyclic voltammetry scan rates performed on the sensors. Outer sphere potentials are provided in (**a**,**b**), whilst inner sphere scans are shown in (**c**,**d**). Second-order best fit polynomials are shown for (**a**,**c**) peak separation, whilst linear fits (with R^2^ > 0.99 for all cases) are shown for the (**b**,**d**) current versus square root of the scan rate.

An increased scan rate, as presented in Figure 8, showed in all cases an increase in the peak separation and peak current. The linearity, as demonstrated in Figure 8b,d, indicates that the redox species is freely diffusing, whilst the increasing peak separation, as shown in Figure 8a,c, suggests that this is an electrochemical quasi-reversible process.

### 3.5. Discussion

The laser heating approach was effective at reducing the graphene oxide, removing between 55% and 75% of the oxygen content, with the remaining content mainly consisting of C-O bonding. Although the performances were generally similar across all four ambient atmospheric conditions, with the vacuum conditions demonstrating a slightly higher quality graphene production with the lowest D/G ratio, the highest 2D/G ratio with a relatively higher oxygen content was found whilst under nitrogen fabrication, and the graphene appeared to be of slightly poorer quality but with the lowest oxygen content.

Outer sphere redox species are insensitive to surface oxides, and this was demonstrated in the cyclic voltammetry results, where all sensors (considering the minimum obtained values) performed well, coming close to the peak-to-peak separation potential limit of 59 mV for the ideal Nernstian one-electron transfer process. Inner sphere redox potentials are sensitive to oxides, which generally leads to a slower transfer rate in all cases. In this work, the better reduction of the oxygen content, as demonstrated by the nitrogen-produced devices, showed the better performance in this respect, achieving a minimum of 66 mV for the peak separation. It is important to note that edge functionality is important for achieving a route for electron transfer, as demonstrated by Griffiths et al. [17], who showed poor electrochemical performance for high-quality, single-layer graphene. This possibly explains the relatively high ΔE_p_ values of the higher quality graphene sensors produced under vacuum conditions. The optimisation of this defect density is required to obtain the best performance from such electrochemical sensors.

Using Equations (A2) and (A3) from Appendix B, the electron transfer rate at the electrode surface may be determined. For the inner sphere redox species potassium ferricyanide, the diffusion coefficient, D, of the oxidation of the electroactive species is approximated to be 5.4 × 10^−6^ cm^2^s^−1^ [65,66]. The results are shown in Table 2 and compared with published work on comparable carbon-based materials. The electrodes produced here are generally comparable to edge plane pyrolytic graphite electrodes but with an optimised fabrication route and have the potential to exceed 0.01 cms^−1^ electron transfer rates, making them comparable to the performance of sensors developed by other groups utilising graphene-based materials.

Previous work has demonstrated that best performing laser-scribed graphene-based electrochemical sensors can achieve electron transfer rates of 23.7 × 10^−3^ cms^−1^ [17]. The main area of difficulty in producing the sensors reported here, as well as an issue for scaling up the procedure, is the reproducibility of the sensor’s fabrication. Scanning electron microscope images, as shown in Figure 2, highlight the problem of the variability in the heating of the surface of the graphene oxide, where it can be seen that the black rGO is interspersed with regions of lighter coloured GO material. The relatively low positional accuracy of the LightScribe system is the root cause of this issue, and this, ultimately, leads to a relatively wide distribution of characterisation and sensor performance results. Future work will look at utilising a laser-heating system with better positional repeatability.

## 4. Conclusions

This work investigated, for the first time, the effect of the laser reduction of graphene oxide using a LightScribe system under a variety of ambient atmospheres producing sensors with applications towards electrochemical sensing. The various atmospheres during reduction led to intriguing initial results, with the differing conditions resulting in varying levels of graphene quality and composition.

Generally speaking, the results demonstrate that an oxygen content of less than 20% is required before a reasonable performance is achieved from the electrodes. The work also highlights a couple of curious results. Firstly, although the sensors produced in air and argon showed comparable results according to the Raman measurements of the graphene reduction and the XPS measurements of the oxygen removal, the inner and outer potentials for the sensors produced in air were significantly poorer than those produced in argon. The only measured difference between these two materials was the type of oxygen bonding present, with those produced in air demonstrating more carboxyl groups present. As outer sphere potentials are insensitive to surface oxides, a possible explanation is a poorer conductivity of the material. Future work will look to clarify this effect with four-point probe measurements on the reduced material to assess the conductivity. Secondly, the sensors produced under vacuum performed surprisingly poorly in the cyclic voltammetry measurements despite showing a comparable oxygen removal to the sensors produced in other atmospheres. These sensors did demonstrate the highest reduction in terms of graphene, with the highest 2D/G ratio and lowest D/G ratio. This implies that a reasonable defect density is required in such reduced graphene electrodes for them to perform well as electrochemical sensors.

The main issue that still needs to be addressed in this work is the repeatability of the fabrication of the sensors, and future work will look to further explore the effect of the ambient atmosphere once fabrication repeatability is established. This work demonstrated, however, that when such sensors are fabricated well, they exhibit good suitability for application in electrochemistry, with electron transfer rates approaching 0.01 cms^−1^ being demonstrated. With both the material and fabrication being cost effective, there are applications in areas requiring large quantities of disposable sensors, such as in biosensing and environmental monitoring.

## Figures and Tables

**Table 1:** Outer and inner sphere potentials for the sensors produced under a specific ambient atmosphere; n = 3 for each atmosphere. The standard deviation is indicated by the error.

Atmosphere	Outer Sphere ΔEp (mV)		Inner Sphere ΔEp (mV)	
	Average ± SD	Lowest Achieved	Average ± SD	Lowest Achieved
Air	142 ± 70	73	153 ± 70	88
Vacuum	125 ± 99	63	181 ± 78	129
Nitrogen	63 ± 1	61	83 ± 15	66
Argon	67 ± 3	63	95 ± 10	85

**Table 2:** Electron transfer rates for carbon-based electrochemical sensors in potassium ferricyanide.

Electrode	k_0_ (×10^−3^ cms^−1^)		Reference
	Average	Best	
Laser-scribed graphene in air	0.50	2.33	This work
Laser-scribed graphene under vacuum	0.31	0.77	This work
Laser-scribed graphene in nitrogen	2.87	10.5	This work
Laser-scribed graphene in argon	1.80	2.57	This work
Edge plane pyrolytic graphite	2.60		[17]
Basal plane pyrolytic graphite	0.33		[17]
Laser-scribed graphene in air	23.7		[17]
Laser-activated graphite screen-printed electrodes	8		[67]
Edge plane pyrolytic graphite	4.66		[68]
Q-graphene modified-edge plane pyrolytic graphite	18.6		[68]

## Data Availability

The data presented in this study are openly available at data.ncl with https://doi.org/10.25405/data.ncl.23803962 (accessed on 29 July 2023).

## Appendix A. Binding Energies

**Table A1:** Binding energies of the expected elements to be found in a survey scan of the sample, with gold being used as an energy calibration standard, and the expected deconvolution components of the carbon peak.

Survey Scan [57]		Carbon Peak [61]	
Regions	Binding Energy (eV)	Regions	Binding Energy (eV)
Au (4f_7/2_, 4f_5/2_)	84.0, 87.7	C-C low	283.4–284.0
C (sp_2_, sp_3_)	284.1, 284.8	C-C, C-H	284.2–284.6
N	400	C-C high	284.8–285.4
O	533	C-O	285.9–286.6
		C=O	286.7–287.5
		O-C=O	288.3–288.9

## Appendix B. Theory of Cyclic Voltammetry

Cyclic voltammetry (CV) is an effective electrochemical method that is commonly used to examine the reduction and oxidation (redox) processes of various molecular species. CV is vital in the study of electron transfer-initiated chemical reactions and has become an important and widely used electroanalytical system in numerous areas of chemistry. The ‘duck-shaped’ plot generated by cyclic voltammetry is a typical electrochemical response observed in a CV experiment. In a representative example [69], the electrochemical process is such that the scan starts at a lower value of potential and then sweeps to higher and more positive oxidative potentials. At the onset of oxidation, the current exponentially increases as the analyte is oxidised at the working electrode surface. Here, the process is under electrochemical control, with the current linearly increasing with increasing voltage until it reaches a peak at a point called the anodic peak current, I_pa_, for the oxidation process at some anodic peak potential, *E_pa_*. From this point onwards in this forward scan, increased potentials slow down the electrochemical process and decrease the current as the potentials are scanned to a more positive potential until a steady state is reached, where further increases in potential no longer affect the observed current. The scan is then reversed to and beyond a potential where the analyte is reduced, with the cathodic peak current, I_pc_, occurring at the cathodic peak potential *E_pc_*.

For the ideal Nernstian one-electron transfer process, the peak-to-peak separation potential, Δ*E_p_*, given by:

(A1) $${\Delta E}_{p}=E_{pa}-E_{pc}$$

is 59 mV [70]. In order to determine the reversibility of a reaction, it is important to determine the electron transfer rate at the electrode surface, *k*_0_. Three regions may be defined for this value [71]:

- *k*_0_ > 500 µms^−1^. The system is regarded as a reversible (or Nerstian) system in which the electron transfer rate is fast enough to sustain the concentrations of the oxidation and reduction electroactive species;
- *k*_0_ < 0.1 µms^−1^. The system is regarded as an irreversible system in which the electron transfer rate is slower than the diffusion constant of the analyte, and so the electrochemically generated species diffuse from the surface of the electrode before a reverse electron transfer reaction can occur;
- 0.1 µms^−1^ < *k*_0_ < 500 µms^−1^. The system is regarded as a quasi-reversible process in which both the electron transfer reaction and the mass transfer process determine the overall rate of the electrode process.

The electron transfer rate is estimated from [72]:

(A2) $$k_{0}=\Psi {\left(\frac{\pi DnvF}{RT}\right)}^{\frac{1}{2}}$$

where *Ψ* is the dimensionless kinetic parameter given by [73]:

(A3) $$\Psi =\frac{\left(0.0021{n\Delta E}_{p}(mV)-0.6288\right)}{\left(1-0.017n\Delta E_{p}(mV)\right)},$$

where *v* is the scan rate (in Vs^−1^), *F* is the Faraday constant, *R* is the universal gas constant, *T* is the absolute temperature, *D* is the diffusion coefficient of the oxidation of the electroactive species and *n* is the number of electrons involved in the electrochemical reaction.

Although there are a multitude of redox reactions that may be assessed with cyclic voltammetry, typically the reduction of ferrocene to ferrocenium and the reduction of ferricyanide to ferrocyanide are used when assessing electrodes. Their solutions are easy to prepare, and with each of these reversible reactions being a single electron process, this allows for the easy interpretation of the cyclic voltagrams produced. The ferrocene reaction is an outer sphere reaction in which electron transfer occurs directly among the complexes. The ferricyanide reaction is an inner sphere reaction in which a bridging ligand is formed to allow for the electron transfer. This process is sensitive to oxides on the electrode surface. For these reasons, these two complexes have become standards for assessing the suitability of materials for use as electrodes.
